# Derivation of All Attitude Error Governing Equations for Attitude Filtering and Control

**DOI:** 10.3390/s19214682

**Published:** 2019-10-28

**Authors:** Ahmad Bani Younes, Daniele Mortari

**Affiliations:** 1 Aerospace Engineering, San Diego State University, 5500 Campanile Drive, San Diego, CA 92182-1308, USA; 2 Aerospace Engineering, Texas A&M University, College Station, TX 77843-3141, USA

**Keywords:** attitude error kinematics, attitude parameterization, Kalman filter

## Abstract

This article presents the full analytical derivations of the attitude error kinematics equations. This is done for several attitude error representations, obtaining compact closed-forms expressions. Attitude error is defined as the rotation between true and estimated orientations. Two distinct approaches to attitude error kinematics are developed. In the first, the estimated angular velocity is defined in the true attitude axes frame, while in the second, it is defined in the estimated attitude axes frame. The first approach is of interest in simulations where the true attitude is known, while the second approach is for real estimation/control applications. Two nonlinear kinematic models are derived that are valid for arbitrarily large rotations and rotation rates. The results presented are expected to be broadly useful to nonlinear attitude estimation/control filtering formulations. A discussion of the benefits of the derived error kinematic models is included.

## 1. Introduction

Many attitude representations are available for modeling problems in science and engineering [1,2,3,4,5,6,7,8,9]. Nonlinearity of the representation of a given physical motion and location of geometric singularities are dependant on (1) the true motion, (2) the attitude representation selected, and (3) the particular choice of estimated axes. Selecting the appropriate representation is highly linked with the kind of problem being considered. The most popular attitude parameterizations are as follows [1,10,11,12]:

- Direction Cosine Matrix (DCM): nine-parameter matrix representation subject to orthogonal constraint, non-singular;
- Principal axis and angle: three-parameter representation (the axis is a unit vector), singular (axis undefined when the angle is zero);
- Euler–Rodrigues parameters (quaternion): four-parameter vector representation subject to unit norm, non-singular;
- Rodrigues parameters (RP; Gibbs vector): three-parameter representation, singular (infinite values for π rotations);
- Modified Rodrigues parameters (MRP): three-parameter representation; and
- Cayley–Klein parameters: 2×2 complex matrix representation subject to unitary constraint.

Definitions, characteristics, and transformations between these representations can be found in many references [1,4,5,6,8,10,13]. For applications requiring large and rapid rotational dynamics, there exists a need for developing attitude error kinematic models that exactly describe arbitrary large rotational motions. In particular, for control problems, Markley [8] has considered different attitude error representations for estimating the state of a maneuvering spacecraft. He has clarified the relationship between the four-component quaternion and the Multiplicative Extended Kalman Filter. Cao et al. [14] developed an unscented predictive filter for satellite formation. Later, Cao et al. [15] proposed a huber-based Kalman filter for the attitude estimation problem of small satellites. Batch and Paielli [16] investigated the rigid-body attitude error using inversion techniques to obtain a robust linearized model of attitude control error dynamics. Sun et al. have derived relative rotational and transnational dynamics error for spacecraft rendezvous control [17]. Crassidis et al. [18] studied a variable-structure control strategy for maneuvering vehicles. In their work, they used a feedback linearizing technique and added an additional term to the spacecraft maneuvers to deal with model uncertainties, which they demonstrated always provides an optimal response. Ahmed et al. [19] extended previous work to consider adaptive asymptotic tracking during maneuvers while estimating inertia properties. They used a Lyapunov argument to generate an unconditionally robust control law with respect to their assumed parametric uncertainty. Bani Younes et al. [20,21] considered generalized optimal control formulations that handle nonlinear system dynamics and enable the development of control gain sensitivities to handle plant model uncertainties during maneuvers. Sharma and Tiwari [22] introduced MRP for parameterization of the orientation error. They defined the attitude error as an additive quantity. Their work is extended by retaining a rigorous nonlinear MRP-based error equation. Schaub et al. [23] developed a new penalty function for optimal control formulation of spacecraft attitude control problems. This function returns the same scalar penalty for a given physical attitude regardless of the attitude coordinate choice. A recent work by Tanygi investigated the projective geometry of vectorial attitude parameterizations with applications for control [24] and estimation [25]. Junkins [26] discussed the link between designing a good controller and the choice of coordinates to represent the attitude kinematics. He linearized the attitude error equations by defining the departure motion as an additive error from a nominal trajectory. Unfortunately, the error in orientation cannot be rigorously represented as additive (linear) because of the nonlinear behavior of underlying kinematical descriptions [2].

Error equations are challenging to define for quaternion variables because of the implied coupling effects between the quaternion components and the implicit norm constraint. The position error is linear and, therefore, can be described by the distance between the two vectors representing the true and the estimated position states. Unfortunately, the error in orientation in not linear and, therefore, must be described by a matrix product (corrective attitude matrix [2]). An exact quaternion error equation is defined as the quaternion product between the true quaternion and the inverse quaternion of the estimated (or reference) state. The significant advantage of this approach is that the norm constraint is preserved and, most importantly, that the quaternion error product remains valid for arbitrarily large relative rotations. This fact is exploited to develop equivalent arbitrarily large relative rotational representations for the vehicle attitude motion. The resulting expressions are compact, accurate, and computationally efficient.

The primary goal of this paper is to develop uniformly valid kinematics equations to describe the time-evolution of the attitude error. References [1,3,4,5,10] contain good review for the attitude kinematics equations. This paper presents compact nonlinear attitude error kinematics equations that can be used in attitude control and/or estimation dynamics problems. Exact analytical (closed-form and no approximations) attitude error kinematic equations are derived for most popular and known attitude representations. This paper extends previous work originally initiated by the authors on developing attitude error kinematics [27,28,29,30], where the estimated angular velocity is defined in the true attitude axes frame. It builds on our initial findings and extends the formulations to address more detailed developments and to present two distinct error kinematics approaches treated with more compact depth and insights in the derivations. It also presents simulation examples that demonstrate the applications in attitude filtering and tracking control. The multiplicative Kalman filter is discussed to enable the use of these attitude error kinematics for two different coordinate choices. The resulting expressions have been optimized to obtain the most compact and computationally efficient forms. Also, the applications of these formulations are discussed by solving the open-loop optimal spacecraft tracking control problem.

The major contribution of this paper is the complete development of attitude error kinematics equations where the estimated angular velocity is defined in either the true attitude axes frame or in the estimated attitude axes frame. These equations can be used for arbitrarily large relative rotations and rotation rates. These attitude errors represent rotations between estimated and true attitudes. Numerical integrations of these kinematic equations are performed to validate the machine error accuracy for each attitude representation. Singularities and constraints are discussed for minimum and non-minimum attitude parameter representations, respectively. Applications are expected in rotational dynamics problems for both nonlinear attitude estimation filtering and attitude tracking.

## 2. Attitude Error Kinematics

**Figure 1:**
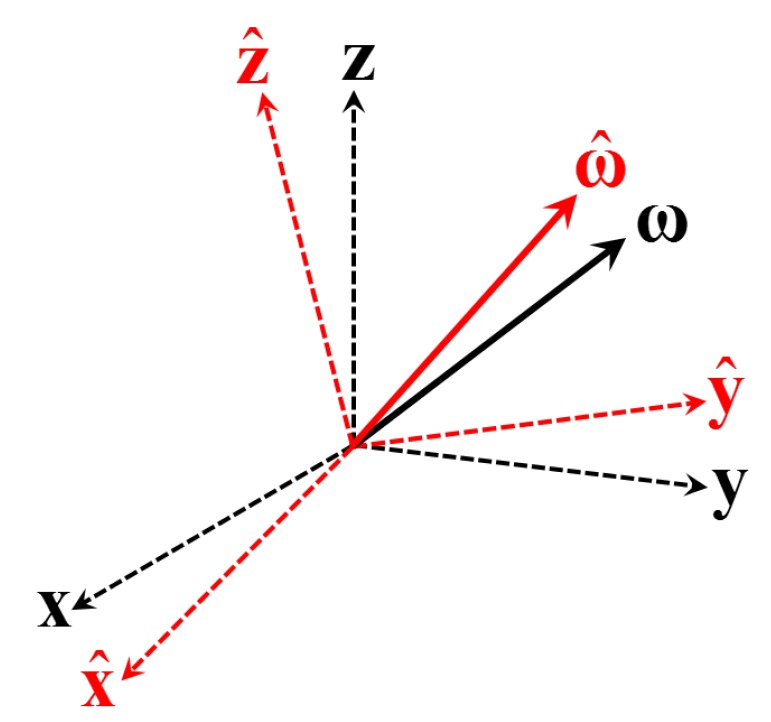
True and estimated ($\hat{•}$) attitude frames and angular velocities

With reference to Figure 1, let *C* be the *true* attitude matrix (DCM) of which the axes are [x,y,z] (dashed, black) and let $\hat{C}$ be the *estimated* attitude matrix of which the axes are $\left[\hat{x},\;\hat{y},\;\hat{z}\right]$ (dashed, red).

The relationships between the DCM with the corresponding Euler’s angles associated with the rotation axes and sequences are given in Table A1. The attitude error is described by the corrective attitude matrix:

(1) $$\delta C=C\;{\hat{C}}^{\;T}.$$

In fact, the product between δC and the estimated attitude $\hat{C}$ provides the true attitude:

$$\delta C\;\hat{C}=\left(C\;{\hat{C}}^{\;T}\right)\;\hat{C}=C.$$

In particular, matrix δC is the transformation matrix for vectors from the estimated $\left[\hat{x},\;\hat{y},\;\hat{z}\right]$ frame to the true [x,y,z] frame.

Let ω be the *true* angular velocity vector (black solid in Figure 1) of which the elements are defined in the [x,y,z] axes of the true attitude. Now, consider $\hat{\omega }$ to be the *estimate* of the true angular velocity vector (red solid in Figure 1). Two distinct cases appears:

(1) The estimated $\hat{\omega }$ is defined in the true body axes frame (x,y,z). In this case, ω and $\hat{\omega }$ are defined in the same reference frame. Therefore, the angular velocity error vector is as follows:
  (2) $$\delta \omega =\omega -\hat{\omega }.$$
  This case finds application in simulations when the true attitude is known.
(2) The estimated $\hat{\omega }$ is defined in the estimated body axes frame ($\hat{x},\;\hat{y},\;\hat{z}$). In this case, ω and $\hat{\omega }$ are defined in two different reference frames. Therefore, the angular velocity error vector is as follows:
  (3) $$\delta \omega =\omega -\delta C\;\hat{\omega },$$
  where δC is provided by Equation (1). This case is important in real applications as the true attitude is always unknown and the estimated angular velocity can be defined in the estimated attitude frame only.

The true attitude dynamics is defined by ω and *C*. They satisfy the DCM kinematic equations:

$$\left[\omega \times \right]=-\dot{C}\;C^{\;T}.$$

The estimated angular velocity satisfies the dynamic equation:

$$I\;\dot{\hat{\omega }}=-\left[\;\hat{\omega }\times \right]\;I\;\hat{\omega }+u,$$

where $I\in {ℜ}^{3\times 3}$ is the moments of inertia tensor and $u\in {ℜ}^{3}$ is the torque acting on the system. We assume that the system parameters are deterministic.

To develop the attitude error kinematics equations, two distinct analysis must be performed. These two derivations are associated to the two angular velocity errors definitions provided in Equations (2) and (3), respectively. The following two sections derive the attitude error kinematics equations valid for arbitrarily large angles and rate errors.

### 2.1. Simulation Case: Estimated Angular Velocity Given in the True Attitude Frame

In this section, the estimated and the true angular velocities are expressed in same coordinate frames, $\delta \omega =\omega -\hat{\omega }$. This approach is suitable in simulations where the true body attitude is known. When using the same reference frame for true and estimated angular velocities, the angular velocity error dynamics equation is written as follows [21,27,28]:

(4) $$\delta \dot{\omega }=-I^{-1}\left\{\left[\;\hat{\omega }\times \right]I-\left[\left(I\hat{\omega }\right)\times \right]\right\}\delta \omega -I^{-1}\left[\left(\delta \omega \right)\times \right]I\delta \omega +I^{-1}u-\dot{\hat{\omega }}-I^{-1}\left[\;\hat{\omega }\times \right]I\hat{\omega }.$$

In the following subsections, the attitude error kinematic equations are derived for the most important attitude representations and for arbitrarily large rotational angles and angular rates. These equations are mathematically simple and compact. They can be used, for instance, to validate novel control theories and/or attitude estimation filtering techniques.

#### 2.1.1. Quaternion Error Kinematics

The quaternion error is a four-dimensional vector defined as follows:

$$\delta q=\left\{\begin{array}{c} \delta q_{v}\\ \delta q_{4} \end{array}\right\},$$

where $\delta q_{v}={\left\{\delta q_{1},\;\delta q_{2},\;\delta q_{3}\right\}}^{T}=e\;\sin\left(\frac{\phi }{2}\right)$, $\delta q_{4}=\cos\left(\frac{\phi }{2}\right)$, e is the principal axis, and ϕ is the principal angle. The kinematic solution for the true quaternion trajectory defines the desired relative rotational motion for the spacecraft. Equation (1) written in term of quaternion is as follows:

(5) $$\delta q=q⊗{\hat{q}}^{-1},$$

where ${\hat{q}}^{-1}$ is the inverse of the estimated quaternion rotation and ⊗ represents the quaternion product. Note that the error, δq, is also a quaternion, that is, a unit vector representing the rotation from the estimated axes to the true axes. This expression is valid for arbitrarily large rational motions and provides a foundation for developing all other kinematic variable generalizations presented. We follow reference [1] in writing the quaternion product as follows:

$$q^{\prime }⊗q=\left\{\begin{array}{c} q_{4}^{\prime }q_{v}+q_{v}^{\prime }q_{4}-q_{v}^{\prime }\times q_{v}\\ q_{4}^{\prime }q_{4}-q_{v}^{\prime }\cdot q_{v} \end{array}\right\},$$

where $q^{\prime }$ and q represent two arbitrary quaternions. The quaternion error rate is as follows:

(6) $$\delta \dot{q}=\dot{q}⊗{\hat{q}}^{-1}+q⊗{\dot{\hat{q}}}^{-1}.$$

Quaternion kinematics evolves according to the kinematic equation:

(7) $$\dot{q}=\frac{1}{2}\left\{\begin{array}{c} \omega \\ 0 \end{array}\right\}⊗q=\frac{1}{2}\left[\begin{array}{cc} -[\;\omega \times ] & \omega \\ -\omega^{T} & 0 \end{array}\right]q=\frac{1}{2}\Omega \left(\omega \right)q.$$

The derivative of the identity $\hat{q}⊗{\hat{q}}^{-1}={\left\{0,0,0,1\right\}}^{T}$ leads to $\dot{\hat{q}}⊗{\hat{q}}^{-1}+\hat{q}⊗{\dot{\hat{q}}}^{-1}=0$. Hence, the inverse quaternion evolves:

(8) $${\dot{\hat{q}}}^{-1}=-\frac{1}{2}{\hat{q}}^{-1}⊗\left\{\begin{array}{c} \hat{\omega }\\ 0 \end{array}\right\}=-\frac{1}{2}\left[\begin{array}{cc} \left[\hat{\omega }\times \right] & \hat{\omega }\\ -{\hat{\omega }}^{T} & 0 \end{array}\right]{\hat{q}}^{-1}=-\frac{1}{2}\Gamma \left(\hat{\omega }\right){\hat{q}}^{-1},$$

where $\Gamma (\hat{\omega })$ is the estimated angular velocity matrix. Substituting Equation (7) and Equation (8) into Equation (6), yields the following:

$$\delta \dot{q}=\frac{1}{2}\Omega \left(\omega \right)\delta q-\frac{1}{2}q⊗{\hat{q}}^{-1}⊗\left\{\begin{array}{c} \hat{\omega }\\ 0 \end{array}\right\}=\frac{1}{2}\Omega \left(\omega \right)\delta q-\frac{1}{2}\delta q⊗\left\{\begin{array}{c} \hat{\omega }\\ 0 \end{array}\right\}.$$

This allows us to write the following:

$$\delta q⊗\left\{\begin{array}{c} \hat{\omega }\\ 0 \end{array}\right\}=\Gamma \left(\hat{\omega }\right)\delta q.$$

The quaternion error rate equation becomes the following:

(9) $$\delta \dot{q}=\frac{1}{2}\left[\Omega \left(\omega \right)-\Gamma \left(\hat{\omega }\right)\right]\delta q.$$

Now, by substituting the angular velocity error given in Equation (2) into Equation (9), the bilinear differential equation for the tracking error kinematics becomes the following:

(10) $$\delta \dot{q}=\frac{1}{2}\left[\Omega \left(\delta \omega +\hat{\omega }\right)-\Gamma \left(\hat{\omega }\right)\right]\delta q=\frac{1}{2}\left[\Omega \left(\delta \omega \right)+\tilde{\Gamma }\left(\hat{\omega }\right)\right]\delta q,$$

where $\tilde{\Gamma }\left(\hat{\omega }\right)$ is a matrix defined as follows:

$$\tilde{\Gamma }\left(\hat{\omega }\right)=\Omega \left(\hat{\omega }\right)-\Gamma \left(\hat{\omega }\right)=\left[\begin{array}{cc} -2[\hat{\omega }\times ] & 0_{3\times 1}\\ 0_{1\times 3} & 0 \end{array}\right].$$

Equation (10) can be rewritten in the following compact form:

(11) $$\delta \dot{q}=\frac{1}{2}\left[\begin{array}{cc} -(\left[\delta \omega \times \right]+2\left[\hat{\omega }\times \right]) & \delta \omega \\ -\delta \omega^{T} & 0 \end{array}\right]\left\{\begin{array}{c} \delta q_{v}\\ \delta q_{4} \end{array}\right\}.$$

This equation can be split into the scalar and vector part of the quaternion as follows [28]:

(12) $$\begin{array}{ccc} \delta {\dot{q}}_{v} & = & \frac{1}{2}\left\{-\left(\left[\delta \omega \times \right]+2\left[\hat{\omega }\times \right]\right)\delta q_{v}+\delta q_{4}\delta \omega \right\}\\ \delta {\dot{q}}_{4} & = & -\frac{1}{2}\delta \omega^{T}\delta q_{v} \end{array}.$$

These above equations are *exact* for arbitrarily large and rapid relative rotational motions.

#### 2.1.2. Rodrigues Parameter Error Kinematics

Rodrigues parameters are a minimum attitude parametrization. The RP vector is defined in terms of quaternion parameters as follows [10]:

(13) $$\rho =\frac{q_{v}}{q_{4}}=e\;\tan\left(\frac{\phi }{2}\right),$$

where the inverse transformation is given as follows:

$$q_{4}=\frac{1}{\sqrt{1+\rho^{2}}}\;\mathrm{and}\;q_{v}=\frac{\rho }{\sqrt{1+\rho^{2}}},$$

where $\rho^{2}=\rho^{T}\rho$. Note that the attitude error given in Equation (12) is represented in the quaternion form. An exact nonlinear model for the quaternions with no approximation is used to preserve the quaternion unit norm. This implies that the quaternion error still represents a finite orientation that can be mapped to any other attitude representations. Here, the quaternion error to RP using Equation (13) is mapped. Thus, the RP error vector is simply expressed as follows:

(14) $$\delta \rho =\frac{\delta q_{v}}{\delta q_{4}}.$$

The inverse mapping for quaternion variables follows:

(15) $$\delta q_{4}=\frac{1}{\sqrt{1+\delta \rho^{2}}}\;\mathrm{and}\;\delta q_{v}=\frac{\delta \rho }{\sqrt{1+\delta \rho^{2}}},$$

where $\delta \rho^{2}=\delta \rho^{T}\delta \rho$. The RP error differential equation is obtained by taking the derivative of Equation (14) and substituting Equations (12) and (15), which yields the following [28]:

(16) $$\delta \dot{\rho }=\frac{1}{2}\left[-\left(\left[\delta \omega \times \right]+2\left[\hat{\omega }\times \right]\right)\delta \rho +\delta \omega \right]+\frac{1}{2}\left(\delta \omega^{T}\delta \rho \right)\delta \rho .$$

Equation (16) provides the desired RP attitude error nonlinear kinematic differential equation.

#### 2.1.3. Modified Rodrigues Parameter Error Kinematics

Modified Rodrigues parameters are an elegant addition to the family of attitude representations. MRP vector is defined in terms of the quaternion parameters by the following [10]

(17) $$\sigma =\frac{q_{v}}{1+q_{4}}=e\;\tan\left(\frac{\phi }{4}\right).$$

The inverse transformation is given by the following:

$$q_{4}=\frac{1-\sigma^{2}}{1+\sigma^{2}}\;\mathrm{and}\;q_{v}=\frac{2\sigma }{1+\sigma^{2}}.$$

Similarly, since the attitude error in Equation (12) is expressed by quaternion, the mapping into MRP can be performed using Equation (17). Thus, MRP error vector is expressed as follows:

(18) $$\delta \sigma =\frac{\delta q_{v}}{1+\delta q_{4}}.$$

The inverse mapping for quaternion variables follows:

(19) $$\delta q_{4}=\frac{1-\delta \sigma^{2}}{1+\delta \sigma^{2}}\;\mathrm{and}\;\delta q_{v}=\frac{2\delta \sigma }{1+\delta \sigma^{2}}.$$

The MRP error differential equation is obtained by taking the derivative of Equation (18) and substituting Equation (12) and Equation (19), which yields the following compact, nonlinear, third-order form [28]:

(20) $$\delta \dot{\sigma }=\frac{1}{4}\left[-2\left(\left[\delta \omega \times \right]+2\left[\hat{\omega }\times \right]\right)\delta \sigma +\left(1-\delta \sigma^{2}\right)\delta \omega \right]+\frac{1}{2}\left(\delta \omega^{T}\delta \sigma \right)\delta \sigma .$$

Equation (20) provides the exact MRP attitude error kinematic differential equation.

#### 2.1.4. Euler Angles Error Kinematics

The most famous attitude representation is described by three angles, known as Euler angles, $\left(\theta_{1},\theta_{2},\theta_{3}\right)$ associated with subsequent rotations about three coordinate axes. The variations of these angles represent the attitude error of δC. There are several conventions for Euler angles, depending on the axes about which the rotations are carried out. In the following, we assume the rotation is 3-1-3 (yaw-roll-yaw) sequences, then we generalize the expression for arbitrary rotation sequence. We start from the mapping equations from quaternion to Euler angles [6]. For the 3-1-3 set, the transformation is as follows:

(21) $$\begin{array}{c} \left\{\begin{array}{c} \delta q_{v}\\ \delta q_{4} \end{array}\right\}={\left\{\begin{array}{c} \sin\left(\frac{\delta \theta_{2}}{2}\right)\;\cos\left(\frac{\delta \theta_{1}-\delta \theta_{3}}{2}\right)\\ \sin\left(\frac{\delta \theta_{2}}{2}\right)\;\sin\left(\frac{\delta \theta_{1}-\delta \theta_{3}}{2}\right)\\ \cos\left(\frac{\delta \theta_{2}}{2}\right)\;\sin\left(\frac{\delta \theta_{1}+\delta \theta_{3}}{2}\right)\\ \cos\left(\frac{\delta \theta_{2}}{2}\right)\;\cos\left(\frac{\delta \theta_{1}+\delta \theta_{3}}{2}\right) \end{array}\right\}}_{313}=\Theta_{313}\left(\delta \theta_{1},\delta \theta_{2},\delta \theta_{3}\right). \end{array}$$

Differentiating Equation (21), we obtain the following:

$$\begin{array}{c} \left\{\begin{array}{c} \delta \dot{q_{v}}\\ \delta \dot{q}4 \end{array}\right\}=H_{313}\left(\delta \theta_{1},\delta \theta_{2},\delta \theta_{3}\right){\left\{\begin{array}{c} \delta {\dot{\theta }}_{1}\\ \delta {\dot{\theta }}_{2}\\ \delta {\dot{\theta }}_{3} \end{array}\right\}}_{313}, \end{array}$$

where $H_{313}\left(\delta \theta_{1},\delta \theta_{2},\delta \theta_{3}\right)$ is a 4×3 matrix. Thus, Euler angle rates can be written in the following least-squares solution:

$$\begin{array}{c} {\left\{\begin{array}{c} \delta {\dot{\theta }}_{1}\\ \delta {\dot{\theta }}_{2}\\ \delta {\dot{\theta }}_{3} \end{array}\right\}}_{313}\;\;\;\;={\left(H_{313}^{T}\;H_{313}\right)}^{-1}\;H_{313}^{T}\left\{\begin{array}{c} \delta \dot{q_{v}}\\ \delta {\dot{q}}_{4} \end{array}\right\}. \end{array}$$

Substituting Equation (12) and making use of Equation (21), we obtain the following:

$$\begin{array}{cc} {\left\{\begin{array}{c} \delta {\dot{\theta }}_{1}\\ \delta {\dot{\theta }}_{2}\\ \delta {\dot{\theta }}_{3} \end{array}\right\}}_{313}\;\;\;\;= & \frac{1}{2}{\left(H_{313}^{T}H_{313}\right)}^{-1}H_{313}^{T}\left[\begin{array}{cc} -(\left[\delta \omega \times \right]+2\left[\hat{\omega }\times \right]) & \delta \omega \\ -\delta \omega^{T} & 0 \end{array}\right]\Theta_{313}\left(\delta \theta_{1},\delta \theta_{2},\delta \theta_{3}\right). \end{array}$$

In general, for the generic *i*-*j*-*k* Euler angles sequence, the formula is as follows [28]:

(22) $$\begin{array}{cc} {\left\{\begin{array}{c} \delta {\dot{\theta }}_{i}\\ \delta {\dot{\theta }}_{j}\\ \delta {\dot{\theta }}_{k} \end{array}\right\}}_{ijk}\;\;\;\;= & \frac{1}{2}{\left(H_{ijk}^{T}H_{ijk}\right)}^{-1}H_{ijk}^{T}\left[\begin{array}{cc} -(\left[\delta \omega \times \right]+2\left[\hat{\omega }\times \right]) & \delta \omega \\ -\delta \omega^{T} & 0 \end{array}\right]\Theta_{ijk}\left(\delta \theta_{1},\delta \theta_{2},\delta \theta_{3}\right). \end{array}$$

Equation (22) represents the attitude error kinematic equation using any Euler angle sequence.

#### 2.1.5. Principal Axis and Angle Error Kinematics

Any rigid-body rotation can be obtained by a single rotation about a principal axis, e, by a principal angle, ϕ. To derive the kinematics of the principal axis/angle for attitude error, we start from the definition of the quaternion:

(23) $$\delta q_{4}=\cos\left(\delta \phi /2\right)\;\mathrm{and}\;\delta q_{v}=\delta e\sin\left(\delta \phi /2\right).$$

Taking the derivative of Equation (23), substituting Equation (12), and solving for $\delta \dot{\phi }$ and $\delta \dot{e}$, we obtain the following [28]:

(24) $$\delta \dot{\phi }=\delta \omega^{T}\delta \hat{e},$$

where $\delta \hat{e}=\frac{\delta e}{\sqrt{\delta e^{T}\delta e}}$ and

(25) $$\delta \dot{e}=-\frac{1}{2}\left(\left(\left[\delta \omega \times \right]+2\left[\hat{\omega }\times \right]\right)\delta \hat{e}+\left[\left(\delta \omega^{T}\delta \hat{e}\right)\delta \hat{e}-\delta \omega \right]\cot\left(\delta \phi /2\right)\right).$$

Equations (24) and (25) are the desired nonlinear kinematic differential equation of the attitude error using principal axis and principal angle.

#### 2.1.6. Direction Cosine Matrix Error Kinematics

The DCM error can be written as follows:

(26) $$\delta C=C\;{\hat{C}}^{\;T}.$$

Performing the derivative of Equation (26) and making use of the attitude kinematics, $\;\dot{C}\;=\;-\;\left[\;\omega \times \right]\;\;\;C$, and the DCM inverse identity, ${\dot{\hat{C}}}^{\;T}=-{\hat{C}}^{\;T}\;\dot{\hat{C}}\;{\hat{C}}^{\;T}$, we obtain the following [28]:

(27) $$\delta \dot{C}=-\left[\delta \omega \times \right]\delta C-\left[\hat{\omega }\times \right]\delta C+\delta C\left[\hat{\omega }\times \right].$$

#### 2.1.7. Cayley–Klein Error Parameters Kinematics

Cayley–Klein parameters is an attitude representation provided by a 2×2 complex matrix. This matrix is as follows:

(28) $$\delta K=\delta q_{4}\;I_{2\times 2}+i\left(\delta q_{1}\sigma_{1}+\delta q_{2}\sigma_{2}+\delta q_{3}\sigma_{3}\right)=\left[\begin{array}{cc} \delta q_{4}+i\delta q_{3} & \delta q_{2}+i\delta q_{1}\\ -\delta q_{2}+i\delta q_{1} & \delta q_{4}-i\delta q_{3} \end{array}\right],$$

where $\sigma_{1}=\left[\begin{array}{cc} 0 & 1\\ 1 & 0 \end{array}\right]$, $\sigma_{2}=\left[\begin{array}{cc} 0 & i\\ -i & 0 \end{array}\right]$, and $\sigma_{3}=\left[\begin{array}{cc} 1 & 0\\ 0 & -1 \end{array}\right]$ are the three Pauli spin matrices. The principal angle can be computed from the following:

$$\delta \phi =2\cos^{-1}\left[\frac{1}{2}\mathrm{tr}\left(\delta K\right)\right].$$

Rewriting Equation (28) in column form, we obtain the following:

(29) $$\begin{array}{c} \mathrm{col}\left(\delta K\right)=\left\{\begin{array}{c} \delta K_{1,1}\\ \delta K_{2,1}\\ \delta K_{1,2}\\ \delta K_{2,2} \end{array}\right\}=\left\{\begin{array}{c} \delta q_{4}+i\delta q_{3}\\ -\delta q_{2}+i\delta q_{1}\\ \delta q_{2}+i\delta q_{1}\\ \delta q_{4}-i\delta q_{3} \end{array}\right\}=\left[\begin{array}{cccc} 0 & 0 & i & 1\\ i & -1 & 0 & 0\\ i & 1 & 0 & 0\\ 0 & 0 & -i & 1 \end{array}\right]\left\{\begin{array}{c} \delta q_{v}\\ \delta q_{4} \end{array}\right\}=\Psi_{0}\delta q, \end{array}$$

where $\Psi_{0}$ is a non-singular constant matrix. Differentiating Equation (29), substituting Equation (12), and using $q_{v}=\Psi_{0}^{-1}\mathrm{col}\left(\delta K\right)$, we obtain the following [28]:

(30) $$\mathrm{col}\left(\delta \dot{K}\right)=\frac{\Psi_{0}}{2}\left[\begin{array}{cc} -\left[\delta \omega \times \right]+2\left[\hat{\omega }\times \right] & \delta \omega \\ -\delta \omega^{T} & 0 \end{array}\right]\Psi_{0}^{-1}\mathrm{col}\left(\delta K\right),$$

where the col(δK) has to satisfy the constraint equation $\delta q^{T}\delta q=1$ that leads to $\mathrm{col}{\left(\delta K\right)}^{T}\Psi_{0}^{-T}\Psi_{0}^{-1}\mathrm{col}\left(\delta K\right)=1$ or $\mathrm{col}{\left(\delta K\right)}^{T}\mathrm{col}\left(\delta K\right)=2$.

### 2.2. Estimation/Control Case: Angular Velocity Estimated in the Estimated Attitude Frame

In this section, the estimated and the true angular velocities are expressed in different coordinate frames: $\delta \omega =\omega -\delta C\hat{\omega }$.

This equation explicitly accounts for the obvious truth that the estimated and true angular velocity vectors are expressed in different coordinate frames. This definition explicitly computes the angular velocity error in the current body frame. This kinematic variable definition leads to the following angular velocity error dynamics equation:

(31) $$\begin{array}{cc} \delta \dot{\omega }= & -I^{-1}\left(\left[\delta C\hat{\omega }\times \right]I-\left[I\delta C\hat{\omega }\times \right]\right)\delta \omega -I^{-1}\left[\delta \omega \times \right]I\delta \omega +I^{-1}u+\\ \; & +\left[\delta \omega \times \right]\delta C\hat{\omega }-\dot{\hat{\omega }}-I^{-1}\left[\delta C\hat{\omega }\times \right]I\delta C\hat{\omega }. \end{array}$$

It is clear that the angular velocity error dynamics equation is coupled with the attitude solution. This is expected as the estimated angular velocity has to be mapped to the true frame using the attitude corrective matrix, δC.

#### 2.2.1. Direction Cosine Matrix Error Kinematics

To investigate the new attitude error parametrization for the estimation/control case, the direction cosine matrix is differentiated for the attitude error given in Equation (1):

(32) $$\delta C=C\;{\hat{C}}^{\;T}\;\to \;\delta \dot{C}=\dot{C}{\hat{C}}^{T}+C{\dot{\hat{C}}}^{T}.$$

Recall the attitude kinematics of the current motion $\dot{C}=-\left[\;\omega \times \right]C$ and the estimated motion $\dot{\hat{C}}=-\left[\;\hat{\omega }\times \right]\hat{C}$. Equation (32) can be written:

(33) $$\delta \dot{C}=-\left[\;\omega \times \right]\delta C+\delta C\left[\;\hat{\omega }\times \right].$$

The angular velocity error is $\delta \omega =\omega -\delta C\hat{\omega }$. Therefore, we obtain the following:

(34) $$\delta \dot{C}=-\left[\delta \omega \times \right]\delta C-\left[\delta C\hat{\omega }\times \right]\delta C+\delta C\left[\;\hat{\omega }\times \right].$$

Using the transformation of skew-symmetric tensor identity [4], we obtain the following:

(35) $$\left[\delta C\hat{\omega }\times \right]=\delta C\left[\;\hat{\omega }\times \right]\delta C^{T}.$$

Also, since δC is an orthogonal matrix, Equation (34) reduces to the following:

(36) $$\delta \dot{C}=-\left[\delta \omega \times \right]\delta C.$$

It is interesting that the attitude error kinematics equation is similar to the attitude kinematics equation. This result agrees with the demonstration presented in Tanygin’s work [24]. Hence, the attitude error kinematics using other attitude parameterizations can be simply obtained.

#### 2.2.2. Quaternion Error Kinematics

The quaternion error is a four-dimensional vector defined as follows:

$$\delta q=\left\{\begin{array}{c} \delta q_{v}\\ \delta q_{4} \end{array}\right\}=\left\{\begin{array}{c} \delta q_{1}\\ \delta q_{2}\\ \delta q_{3}\\ \delta q_{4} \end{array}\right\}.$$

The transformation from quaternion error, δq, to DCM error, δC, is given by the following:

(37) $$\delta C=\left[\begin{array}{c} \left(\delta q_{4}^{2}-\delta q_{v}^{T}\delta q_{v}\right)I+2\delta q_{v}\delta q_{v}^{T}-2\delta q_{4}\left[\delta q_{v}\times \right] \end{array}\right].$$

The inverse transformations is as follows:

(38) $$\begin{array}{c} \delta q_{4}=\pm \frac{1}{2}\sqrt{1+\mathrm{tr}[\delta C]} \end{array}$$

(39) $$\begin{array}{c} \delta q_{v}=\frac{1}{4\delta q_{4}}\left\{\begin{array}{c} \delta C_{23}-\delta C_{32}\\ \delta C_{31}-\delta C_{13}\\ \delta C_{12}-\delta C_{21} \end{array}\right\}. \end{array}$$

This transformation shows the redundancy in selecting the sign of the scalar parameter, $\delta q_{4}$. The positive sign is associated to δϕ≤π, while the negative is associated to π<δϕ≤2π.

The kinematics differential equation of the quaternion error is derived by differentiating Equation (38):

(40) $$\begin{array}{c} \delta {\dot{q}}_{4}=\frac{\delta {\dot{C}}_{11}+\delta {\dot{C}}_{22}+\delta {\dot{C}}_{33}}{8\delta q_{4}}. \end{array}$$

Substituting the expressions of the diagonal elements

$$\begin{array}{cc} \delta {\dot{C}}_{11}= & -\left(\delta C_{23}-\delta C_{32}\right)\delta \omega_{1}\\ \delta {\dot{C}}_{22}= & -\left(\delta C_{31}-\delta C_{13}\right)\delta \omega_{2}\\ \delta {\dot{C}}_{33}= & -\left(\delta C_{12}-\delta C_{21}\right)\delta \omega_{3} \end{array}$$

in Equation (40) yields the following:

(41) $$\delta {\dot{q}}_{4}=\frac{1}{2}\left(-\delta q_{1}\delta \omega_{1}-\delta q_{2}\delta \omega_{2}-\delta q_{3}\delta \omega_{3}\right)=-\frac{1}{2}\delta q_{v}^{T}\delta \omega .$$

Similar derivations are applied on $\delta {\dot{q}}_{1}$, $\delta {\dot{q}}_{2}$, and $\delta {\dot{q}}_{3}$ in Equation (). The kinematic error differential equation for quaternion is then the following:

(42) $$\delta \dot{q}=\frac{1}{2}\left[\begin{array}{c} \delta q_{4}I_{3\times 3}+\left[\;\delta q_{v}\times \right]\\ -\delta q_{v}^{T} \end{array}\right]\delta \omega =\frac{1}{2}\left[\begin{array}{cc} -[\;\delta \omega \times ] & \delta \omega \\ -\delta \omega^{T} & 0 \end{array}\right]\delta q,$$

where $I_{3\times 3}$ is the 3×3 identity matrix.

#### 2.2.3. Rodrigues Parameter Error Kinematics

Rodrigues parameters error vector is simply expressed by the following:

(43) $$\delta \rho =\frac{\delta q_{v}}{\delta q_{4}}=\tan\left(\frac{\delta \phi }{2}\right)\delta e,$$

Also, the inverse mapping for quaternion variables follow:

(44) $$\delta q_{4}=\frac{1}{\sqrt{1+\delta \rho^{2}}}\;\mathrm{and}\;\delta q_{v}=\frac{\delta \rho }{\sqrt{1+\delta \rho^{2}}},$$

where $\delta \rho^{2}=\delta \rho^{T}\delta \rho$. The differential equation for the RP error follows by taking the derivative of Equation (43):

(45) $$\delta \dot{\rho }=\frac{\delta \dot{q_{v}}}{\delta q_{4}}-\frac{\delta {\dot{q}}_{4}\delta q_{v}}{{\left(\delta q_{4}\right)}^{2}}.$$

Substituting Equation (42) into Equation (45) yields the following:

$$\delta \dot{\rho }=\frac{1}{2}\left[\frac{\delta q_{4}I_{3\times 3}+\left[\;\delta q_{v}\times \right]}{\delta q_{4}}+\frac{\delta q_{v}}{{\left(\delta q_{4}\right)}^{2}}\delta q_{v}^{T}\right]\delta \omega .$$

This equation can be further simplified by substituting Equation (44) to yield the compact quadratic form:

(46) $$\delta \dot{\rho }=\frac{1}{2}\left[I_{3\times 3}+\left[\;\delta \rho \times \right]+\delta \rho \delta \rho^{T}\right]\delta \omega .$$

Equation (46) is the RP attitude error kinematic differential equation. The above form approaches singularity when |δϕ|→±π.

#### 2.2.4. Modified Rodrigues Parameter Error Kinematics

Modified Rodrigues parameters error vector is expressed as follows:

(47) $$\delta \sigma =\frac{\delta q_{v}}{1+\delta q_{4}}=\tan\left(\frac{\delta \phi }{4}\right)\delta e,$$

The inverse mapping to quaternion is as follows:

(48) $$\delta q_{4}=\frac{1-\delta \sigma^{2}}{1+\delta \sigma^{2}}\;\mathrm{and}\;\delta q_{v}=\frac{2\delta \sigma }{1+\delta \sigma^{2}}.$$

The differential equation for the MRP error follows from the derivative of Equation (47):

(49) $$\delta \dot{\sigma }=\frac{\delta \dot{q_{v}}}{1+\delta q_{4}}-\frac{\delta {\dot{q}}_{4}\delta q_{v}}{{\left(1+\delta q_{4}\right)}^{2}}.$$

Substituting Equation (42) into Equation (49) yields the following:

$$\delta \dot{\sigma }=\frac{1}{2}\left[\frac{\delta q_{4}I_{3\times 3}+\left[\;\delta q_{v}\times \right]}{1+\delta q_{4}}+\frac{\delta q_{v}\delta q_{v}^{T}}{{\left(1+\delta q_{4}\right)}^{2}}\right]\delta \omega .$$

This equation is simplified by substituting Equation (48) to obtain a compact quadratic form:

(50) $$\delta \dot{\sigma }=\frac{1}{4}\left[\left(1-\delta \sigma^{2}\right)I_{3\times 3}+2\left[\delta \sigma \times \right]+2\delta \sigma^{T}\delta \sigma \right]\delta \omega .$$

Equation (50) provides the MRP attitude error kinematic differential equation. The above form approaches singular behavior as |δϕ|→±2π.

#### 2.2.5. Euler Angles Error Kinematics

There are 12 distinct Euler angles sequences, depending on the axes about which the rotations are carried out. Review the development in Section 2.1.4; similar derivations are followed here that yield to the general *i*-*j*-*k* Euler angle error kinematics equations:

(51) $$\begin{array}{cc} {\left\{\begin{array}{c} \delta {\dot{\theta }}_{i}\\ \delta {\dot{\theta }}_{j}\\ \delta {\dot{\theta }}_{k} \end{array}\right\}}_{ijk}\;\;\;\;= & \frac{1}{2}{\left(H_{ijk}^{T}H_{ijk}\right)}^{-1}H_{ijk}^{T}\left[\begin{array}{cc} -[\delta \omega \times ] & \delta \omega \\ -\delta \omega^{T} & 0 \end{array}\right]\Theta_{ijk}\left(\delta \theta_{1},\delta \theta_{2},\delta \theta_{3}\right). \end{array}$$

Equation (51) is the desired kinematic differential equation using Euler angles to represent the attitude error.

Alternatively, the Euler angles error kinematics can be derived by writing the angular velocity error vector in the body frame in terms of the Euler angles error rates. For example, the angular velocity error vector for 3-1-3 rotation sequence is given by the following:

(52) $$\delta \omega =R_{313}\left(\delta \theta_{1},\delta \theta_{2},\delta \theta_{3}\right)\left\{\begin{array}{c} 0\\ 0\\ \delta {\dot{\theta }}_{1} \end{array}\right\}+R_{31}\left(\delta \theta_{2},\delta \theta_{3}\right)\left\{\begin{array}{c} \delta {\dot{\theta }}_{2}\\ 0\\ 0 \end{array}\right\}+R_{3}\left(\delta \theta_{3}\right)\left\{\begin{array}{c} 0\\ 0\\ \delta {\dot{\theta }}_{3} \end{array}\right\},$$

where $R_{313}\left(\delta \theta_{1},\delta \theta_{2},\delta \theta_{3}\right)=R_{3}\left(\delta \theta_{3}\right)R_{1}\left(\delta \theta_{2}\right)R_{3}\left(\delta \theta_{1}\right)$ is the 3-1-3 rotation sequence, where

(53) $$\begin{array}{cc} R_{1}\left(\delta \theta \right)= & \left[\begin{array}{ccc} 1 & 0 & 0\\ 0 & \cos(\delta \theta ) & \sin(\delta \theta )\\ 0 & -\sin(\delta \theta ) & \cos(\delta \theta ) \end{array}\right],\\ R_{2}\left(\delta \theta \right)= & \left[\begin{array}{ccc} \cos(\delta \theta ) & 0 & -\sin(\delta \theta )\\ 0 & 1 & 0\\ \sin(\delta \theta ) & 0 & \cos(\delta \theta ) \end{array}\right],\;\mathrm{and}\\ R_{3}\left(\delta \theta \right)= & \left[\begin{array}{ccc} \cos(\delta \theta ) & -\sin(\delta \theta ) & 0\\ \sin(\delta \theta ) & \cos(\delta \theta ) & 0\\ 0 & 0 & 1 \end{array}\right]. \end{array}$$

Equation (52) can be written in the compact form:

(54) $$\delta \omega =M_{313}\left\{\begin{array}{c} \delta {\dot{\theta }}_{1}\\ \delta {\dot{\theta }}_{2}\\ \delta {\dot{\theta }}_{3} \end{array}\right\},$$

where

$$M_{313}=\left[\begin{array}{ccc} \sin\left(\delta \theta_{2}\right)\sin\left(\delta \theta_{3}\right) & \cos(\delta \theta_{3}) & 0\\ \sin\left(\delta \theta_{2}\right)\cos\left(\delta \theta_{3}\right) & -\sin(\delta \theta_{3}) & 0\\ \cos(\delta \theta_{2}) & 0 & 1 \end{array}\right].$$

The kinematic differential equation of 3-1-3 Euler angles error is the inverse of Equation (54):

(55) $$\left\{\begin{array}{c} \delta {\dot{\theta }}_{1}\\ \delta {\dot{\theta }}_{2}\\ \delta {\dot{\theta }}_{3} \end{array}\right\}=M_{313}^{-1}\left\{\begin{array}{c} \delta \omega_{1}\\ \delta \omega_{2}\\ \delta \omega_{3} \end{array}\right\}=M_{313}^{-1}\;\delta \omega .$$

The complete set of 12 Euler angles error kinematics in terms of $M_{ijk}^{-1}$ and the angular velocity error vector is provided in Appendix A.

#### 2.2.6. Principal Axis and Angle Error Kinematics

To derive the kinematics of the principal axis/angle for attitude error, we start from the definition of the quaternion:

$$\delta q_{4}=\cos\left(\delta \phi /2\right)\;\mathrm{and}\;\delta q_{v}=\delta e\sin\left(\delta \phi /2\right).$$

Taking the derivative and solving for $\delta \dot{\phi }$ and $\delta \dot{e}$, we obtain the following:

$$\delta \dot{\phi }=-\frac{2\;\delta {\dot{q}}_{4}}{\sin(\delta \phi /2)}\;\mathrm{and}\;\delta \dot{e}=\frac{\delta {\dot{q}}_{v}-\frac{1}{2}\delta e\delta \dot{\phi }\cos\left(\delta \phi /2\right)}{\sin(\delta \phi /2)}.$$

Substituting Equation (42) and making use of $\delta q_{v}=\delta \hat{e}\;\sin\left(\delta \phi /2\right)$ and the identity $\left[\delta \;\hat{e}\times \right]\;\left[\delta \;\hat{e}\times \right]\;=\;\delta \hat{e}\delta {\hat{e}}^{T}-I_{3\times 3}$, we obtain the following:

(56) $$\delta \dot{\phi }=\delta \omega^{T}\delta \hat{e},$$

where $\delta \hat{e}=\frac{\delta e}{\sqrt{\delta e^{T}\delta e}}$ and

(57) $$\delta \dot{e}=\frac{1}{2}\left\{\left[\delta \hat{e}\times \right]-\left[\delta \hat{e}\times \right]\left[\delta \hat{e}\times \right]\;\cot\left(\delta \phi /2\right)\right\}\;\delta \omega .$$

Equations (56) and (57) represent the kinematic equations of the attitude error using principal axis and principal angle.

#### 2.2.7. Cayley–Klein Error Parameters Kinematics

Cayley–Klein parameters is an attitude representation provided by a 2×2 complex matrix. This matrix is as follows:

$$\delta K=\delta q_{4}\;I_{2\times 2}+i\left(\delta q_{1}\sigma_{1}+\delta q_{2}\sigma_{2}+\delta q_{3}\sigma_{3}\right)=\left[\begin{array}{cc} \delta q_{4}+i\delta q_{3} & \delta q_{2}+i\delta q_{1}\\ -\delta q_{2}+i\delta q_{1} & \delta q_{4}-i\delta q_{3} \end{array}\right],$$

where $\sigma_{1}$, $\sigma_{2}$, and $\sigma_{3}$ are the three Pauli spin matrices. The principal angle can be computed from the following:

$$\delta \phi =2\cos^{-1}\left[\frac{1}{2}\mathrm{tr}\left(\delta K\right)\right].$$

It has been already shown in (Section 2.1.7) that the Cayley–Klein parameters can be written in the column form:

$$\begin{array}{c} \mathrm{col}\left(\delta K\right)=\left\{\begin{array}{c} \delta K_{1,1}\\ \delta K_{2,1}\\ \delta K_{1,2}\\ \delta K_{2,2} \end{array}\right\}=\Psi_{0}\delta q, \end{array}$$

where $\Psi_{0}=\left[\begin{array}{cccc} 0 & 0 & i & 1\\ i & -1 & 0 & 0\\ i & 1 & 0 & 0\\ 0 & 0 & -i & 1 \end{array}\right]$ is a non-singular constant matrix. The kinematics equation of the Cayley–Klein error column vector is as follows:

(58) $$\begin{array}{c} \mathrm{col}\left(\delta \dot{K}\right)=\Psi_{0}\delta \dot{q}. \end{array}$$

Substituting Equation (42) into Equation (58) and making use of $\mathrm{col}\left(\delta K\right)=\Psi_{0}\delta q$, the kinematics equation of the Cayley–Klein error column vector yields the following:

(59) $$\mathrm{col}\left(\delta \dot{K}\right)=\frac{1}{2}\Psi_{0}\left[\begin{array}{cc} -[\delta \omega \times ] & \delta \omega \\ -\delta \omega^{T} & 0 \end{array}\right]\Psi_{0}^{-1}\mathrm{col}\left(\delta K\right),$$

where the col(δK) has to satisfy the constraint equation $\mathrm{col}{\left(\delta K\right)}^{T}\mathrm{col}\left(\delta K\right)=2$.

Appendix B summarizes the attitude error kinematics using the two definitions of the angular velocity error.

### 2.3. Numerical Validation

**Figure 2:**
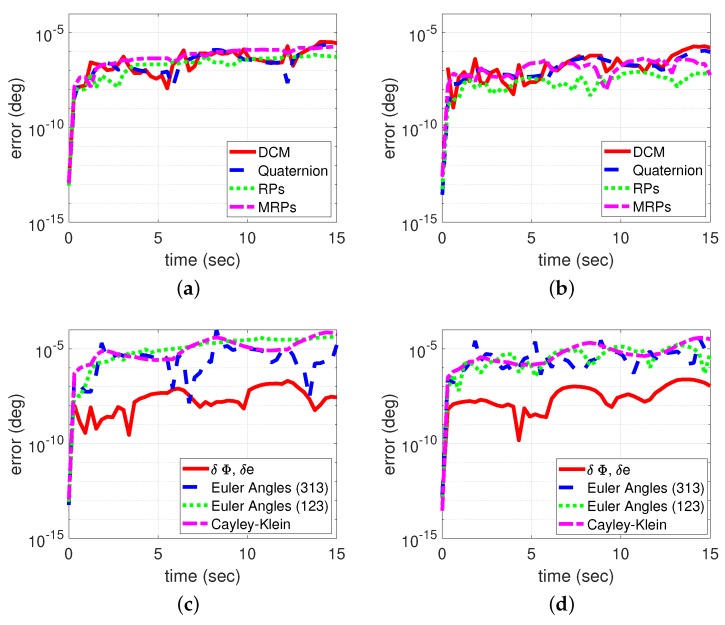
Validation test. (large angular rotation δϕ=[145,170] deg). (**a**) Kinematics error 1. (**b**) Kinematics error 2. (**c**) Kinematics error 1. (**d**) Kinematics error 2. DCM= Direction Cosine Matrix; RP= Rodrigues parameters; MRP = Modified Rodrigues parameters.

**Figure A1:**
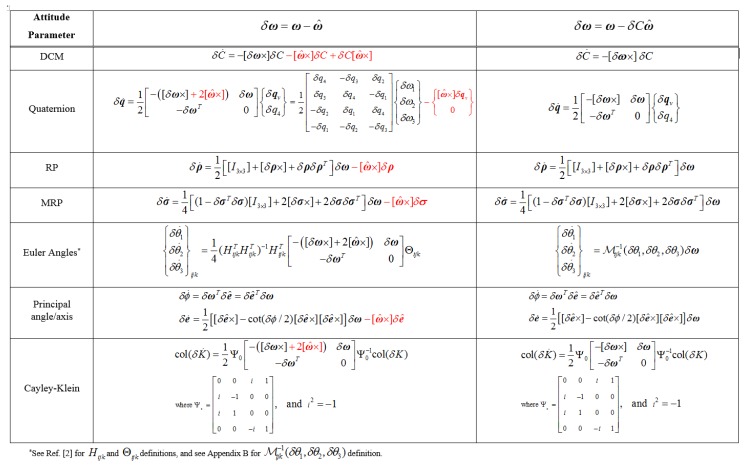
Summary of the attitude error kinematics equations. The terms in red indicate the differences between the two forms

Numerical integrations of all the kinematic equations presented are performed for arbitrary initial conditions to validate the machine error accuracy for each attitude representation. For this particular example, the initial conditions are given in Table 1, where constant angular velocity is considered. The validation test is performed as follows:

1. Integrate the *true* attitude kinematics for each attitude representation using the attitude kinematics equations;
2. Transform the time history solution of the *true* attitude for each attitude representation to the direction cosine matrix *C* or quaternion q;
3. Integrate the *estimated* attitude kinematics for each attitude representation using the attitude kinematics equations;
4. Transform the time history solution of the *estimated* attitude for each attitude representation to the direction cosine matrix $\hat{C}$ or quaternion $\hat{q}$;
5. Calculate the *attitude error* of the two solutions in steps 2 and 4 using $\delta C=C{\hat{C}}^{\;T}$ or $\delta q=q⊗{\hat{q}}^{-1}$; then, calculate the principal angle of the attitude error, $\delta \phi^{*}$;
6. For approaches 1 and 2, integrate the *attitude error kinematics* for each attitude representation using the attitude error kinematics equations in Appendix B (Table Figure A1); then, calculate the principal angle of the attitude error δϕ for each approach;
7. Calculate the absolute error $\Delta \left(\delta \phi \right)=|\delta \phi^{*}-\delta \phi |$, as shown in Figure 2.

Figure 2 represents the validation test of the two approaches (step 6) compared to the classical approach (step 5). Figure 2a,b show the computation error, $\Delta \left(\delta \phi \right)=|\delta \phi^{*}-\delta \phi |$, of the attitude error computed using the first approach (Figure 2a) and the second approach (Figure 2b) for the following attitude representations: direction cosine matrix, quaternion, Rodrigues parameters, and modified Rodrigues parameters. The first approach represents the *simulation* case, denoted by kinematic error 1. The second approach represents the*estimation/control* case, denoted by kinematic error 2. The two figures Figure 2c,d show the computation error, $\Delta \left(\delta \phi \right)=|\delta \phi^{*}-\delta \phi |$, of the attitude error computed using the first approach (Figure 2c) and the second approach (Figure 2d) for the following attitude representations: principal axis/angle, Euler angles (3-1-3), Euler angles (1-2-3), and Cayley–Klein parameters. The computation error retains the accuracy level of the numerical integrator used (MATLAB®  function ode45).

## 3. Kalman Filter

In this section, a sequential extended Kalman filter (EKF) formulation is developed and presented for the two approaches. The development of compact forms of nonlinear attitude error kinematics enables the applications of arbitrarily large relative rotations and rotation rates in different attitude coordinates. Since quaternions present no singularities, it is the most popular coordinate for attitude estimation. However, it must obey the norm constraint.

### 3.1. Estimated Angular Velocity Defined in the True Attitude Frame

The estimated angular velocity vector $\hat{\omega }$ is defined in the true body axes frame (x,y,z). In this case, ω and $\hat{\omega }$ are defined in the same reference frame. Therefore, the angular velocity error vector is as follows:

$$\delta \omega =\omega -\hat{\omega }.$$

This case finds application during simulations when the true attitude is available. The quaternion error kinematics is given by Equation (12), which can be linearized for first-order approximation to the following [8]:

(60) $$\begin{array}{ccc} \delta {\dot{q}}_{v} & \approx & -\left[\hat{\omega }\times \right]\delta q_{v}+\frac{1}{2}\delta \omega \\ \delta {\dot{q}}_{4} & \approx & 0. \end{array}$$

Unfortunately, this linearization requires that the estimated quaternion is close to the true quaternion to preserve the normalization constraint.

A common sensor model to measure the angular rate is the rate-integrating gyro, which is defined by the following:

(61) $$\begin{array}{ccc} \omega & = & \tilde{\omega }-\beta -\eta_{v}\\ \dot{\beta } & = & \eta_{u}, \end{array}$$

where $\eta_{v}∼N\left(0,\sigma_{v}^{2}I_{3\times 3}\right)$ and $\eta_{u}∼N\left(0,\sigma_{u}^{2}I_{3\times 3}\right)$ are zero-mean Gaussian noise with variances given by $\sigma_{v}^{2}$ and $\sigma_{u}^{2}$, respectively; β is the gyro bias vector; and $\tilde{\omega }$ is the measured angular velocity, which is given by the following:

(62) $$\tilde{\omega }=\hat{\omega }+\hat{\beta },$$

where $\hat{\omega }$ and $\hat{\beta }$ are the estimated angular velocity and the estimated gyro bias vectors, respectively. Hence, the angular velocity error is given by the following:

(63) $$\delta \omega =-(\delta \beta +\eta_{v}),$$

where $\delta \beta =\beta -\hat{\beta }$. The multiplicative extended Kalman filter error model is now given by the following (note: $\dot{\hat{\beta }}=0$):

(64) $$\begin{array}{ccc} \delta {\dot{q}}_{v} & \approx & -\left[\hat{\omega }\times \right]\delta q_{v}-\frac{1}{2}\left(\delta \beta +\eta_{v}\right)\\ \delta \dot{\beta } & \approx & \eta_{u}. \end{array}$$

### 3.2. Estimated Angular Velocity Defined in the Estimated Attitude Frame

In this section, the estimated and the true angular velocities are expressed in different coordinate frames, $\delta \omega =\omega -\delta C\hat{\omega }$. It has been discussed earlier that the attitude error kinematics equations for this approach take the same form as the attitude kinematics equations; see Figure A1. The quaternion error kinematics is linearized for first-order approximation to the following:

(65) $$\begin{array}{ccc} \delta {\dot{q}}_{v} & \approx & \frac{1}{2}\delta \omega \\ \delta {\dot{q}}_{4} & \approx & 0, \end{array}$$

where the first-order approximation of the attitude error matrix is given by the following:

(66) $$\delta C\approx I_{3\times 3}-2\left[\delta q_{v}\times \right].$$

Considering the same rate-integrating gyro sensor model defined in Equation (61) and the observed angular velocity definition in Equation (62), the angular velocity error is written as follows:

(67) $$\delta \omega =\left(I_{3\times 3}-\delta C\right)\tilde{\omega }-\left(\delta \beta +\eta_{v}\right)=-2\left[\left(\hat{\omega }+\hat{\beta }\right)\times \right]\delta q_{v}-\left(\delta \beta +\eta_{v}\right),$$

where $\delta \beta =\beta -\delta C\hat{\beta }$. The multiplicative extended Kalman filter error model is now given by the following (note: $\dot{\hat{\beta }}=0$):

(68) $$\begin{array}{ccc} \delta {\dot{q}}_{v} & \approx & -\left[\left(\hat{\omega }+\hat{\beta }\right)\times \right]\delta q_{v}-\frac{1}{2}\left(\delta \beta +\eta_{v}\right)\\ \delta \dot{\beta } & \approx & \eta_{u}+\left[\delta \omega \times \right]\delta C\hat{\beta }. \end{array}$$

### 3.3. Extended Kalman Filter Error Model

For a single sensor, we define the true and estimated body vectors as follows:

(69) $$\begin{array}{ccc} b & = & C(q)r\\ {\hat{b}}^{-} & = & C({\hat{q}}^{-})r, \end{array}$$

where $r={\left\{x,\;y,\;z\right\}}^{T}$ is the 3×1 vector of the vehicle position in the earth-centered, earth-fixed (ECEF) coordinates, the true attitude matrix is $C\left(q\right)\;=\;C\;\left(\delta q\right)C\left({\hat{q}}^{-}\right)$, C(δq) is defined in Equation (37), and $C\left(\hat{q}\right)\;=\;\left[\begin{array}{c} \;\left({\hat{q}}_{4}^{2}-{\hat{q}}_{v}^{T}{\hat{q}}_{v}\right)I+2{\hat{q}}_{v}\delta {\hat{q}}_{v}^{T}-2{\hat{q}}_{4}\left[{\hat{q}}_{v}\times \right] \end{array}\right]$. Thus, the estimation error of the body vector is defined as follows:

(70) $$\Delta b\equiv b-{\hat{b}}^{-}=2\left[C\left({\hat{q}}^{-}\right)r\times \right]\delta q_{v}.$$

The sensitivity matrix of *n* measurement sets is given by the following:

(71) $$H=\left[\begin{array}{cc} 2[C\left({\hat{q}}^{-}\right)r_{1}\times ] & 0_{3\times 3}\\ 2[C\left({\hat{q}}^{-}\right)r_{2}\times ] & 0_{3\times 3}\\ \vdots & \vdots \\ 2[C\left({\hat{q}}^{-}\right)r_{n}\times ] & 0_{3\times 3} \end{array}\right].$$

The EKF error model is given by the following:

(72) $$\Delta \dot{\tilde{x}}=F\left(\hat{x}\left(t\right),t\right)\Delta \tilde{x}\left(t\right)+G\left(t\right)w\left(t\right),$$

where $\Delta \tilde{x}\left(t\right)={\left[\delta q_{v}^{T}\;\delta \beta^{T}\right]}^{T};\;w={\left[\eta_{v}^{T}\;\eta_{u}^{T}\right]}^{T}$; and $F(\hat{x}\left(t\right),t),\;G\left(t\right)$, and Q(t) are given by Table 2:

Discrete-time attitude observation n×1 model at time $t_{k}$ is given by the following:

(73) $${\tilde{y}}_{k}={\left\{\begin{array}{c} \left[C\left(q\right)r_{1}\right]\\ q)r_{2}]\\ \vdots \\ q)r_{n}] \end{array}\right\}}_{t_{k}}+{\left\{\begin{array}{c} \nu_{1}\\ \nu_{2}\\ \vdots \\ \nu_{n} \end{array}\right\}}_{t_{k}}\equiv h_{k}\left({\hat{x}}_{k}\right)+v_{k},$$

where $v_{k}∼N\left(0,R_{k}\right)$ is zero-mean gaussian measurements noise with covariance error matrix $R_{k}$. Thus, the error state update is given by the following:

(74) $$\Delta {\hat{\tilde{x}}}_{k}^{+}=K_{k}\left[{\tilde{y}}_{k}-h_{k}\left({\hat{x}}_{k}^{-}\right)\right],$$

where $\Delta {\hat{\tilde{x}}}_{k}^{+}={\left[\delta {\hat{\tilde{q}}}_{k}^{+}\;\delta {\hat{\tilde{\beta }}}_{k}^{+}\right]}^{T}$, ${\tilde{y}}_{k}$ is the measurement output, $h_{k}\left({\hat{x}}_{k}^{-}\right)$ is the estimate output, and $K_{k}$ is Kalman gain, as given in Equation (75).

The EKF is implemented following the below sequential steps [30,31]:

- *Initialization*: at $t_{0}$ and for given initial states $x_{0}={\left[{\hat{q}}_{0},\;\beta_{0}\right]}^{T}$ and initial value of the covariance matrix $P_{0}$, the initial values are given by the following:
  (75) $$\begin{array}{ccc} {\hat{x}}^{-}\left(0\right) & = & E\left\{x\left(0\right)\right\}=x_{0}\\ P^{-}\left(0\right) & = & E\left\{\left(x\left(0\right)-x_{0}\right){\left(x\left(0\right)-x_{0}\right)}^{T}\right\}=P_{0}, \end{array}$$
  where the superscript (−) denotes priori values and E{} is the expectation operator. Assume that x(0)∼N(0,P(0)).
- *Gain*: compute the Kalman gain matrix:
  (76) $$K_{k}=P_{k}^{-}H_{k}^{T}\left({\hat{x}}_{k}^{-}\right){\left[H_{k}\left({\hat{x}}_{k}^{-}\right)P_{k}^{-}H_{k}^{T}\left({\hat{x}}_{k}^{-}\right)+R_{k}\right]}^{-1},$$
  where $H_{k}\left({\hat{x}}_{k}^{-}\right)\equiv \frac{\partial h}{\partial x}{|}_{{\hat{x}}_{k}^{-}}$ is given in Equation (71).
- *Update*: update the state estimate ${\hat{x}}_{k}^{+}$ and covariance $P_{k}^{+}$ at each measurement:
  (77) $$\begin{array}{ccc} {\hat{x}}_{k}^{+} & = & {\hat{x}}_{k}^{-}+K_{k}\left[{\tilde{y}}_{k}-h_{k}\left({\hat{x}}_{k}^{-}\right)\right]\\ P_{k}^{+} & = & \left[I_{3\times 3}-K_{k}H_{k}\left({\hat{x}}_{k}^{-}\right)\right]P_{k}^{+}, \end{array}$$
  where the superscript (+) denotes posteriori values.
- *Propagation*: propagate both the state estimate ${\hat{x}}_{k}$ and covariance $P_{k}$ using the posteriori estimate ${\hat{x}}_{k}^{+}$ and posteriori covariance $P_{k}^{+}$. The estimated angular velocity, $\hat{\omega }=\tilde{\omega }-\hat{\beta }$, is used to propagate the quaternion kinematics:
  (78) $$\begin{array}{ccc} \dot{\hat{q}} & = & \frac{1}{2}\left[\begin{array}{cc} -[\;\omega \times ] & \hat{\omega }\\ -{\hat{\omega }}^{T} & 0 \end{array}\right]\hat{q}\\ \dot{\hat{\beta }} & = & 0\\ \dot{P} & = & FP+PF^{T}+GQG^{T}, \end{array}$$
  where the matrices *F*, *G*, and *Q* are given by Table 2.

### 3.4. Numerical Simulation

This section presents simulation results that utilize data provided by a star tracker to estimate the attitude of slow spinning spacecraft under the following scenario:

- Optical axis aligned with $\hat{z}$ axis of the body reference frame.
- Sensor field of view: $10^{\circ }\times 12^{\circ }$.
- Star catalog with magnitude threshold = 6.
- Observed stars affected by multiplicative Gaussian noise [32] due to centroid error, $3\sigma =20^{\prime \prime }$.
- spacecraft is spinning about the $\hat{y}$ axis with constant angular velocity 1.01 rad/s.
- Simulation time is 300 s with sampling frequency of 10 Hz.

**Figure 3:**
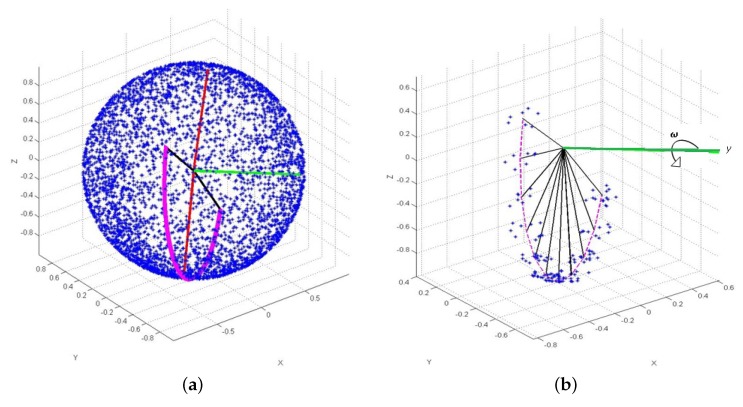
Stars view over the trajectory. (**a**) Stars sphere. (**b**) Observed stars by an orbiting spacecraft.

**Figure 4:**
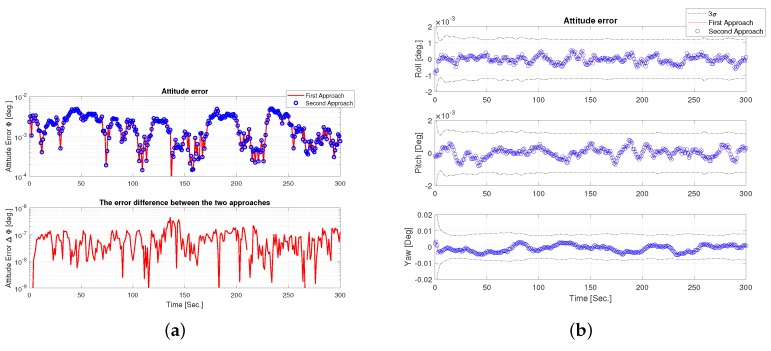
Attitude estimation. (**a**) Attitude estimation error: principal angle. (**b**)Attitude estimation error: Euler’s angles.

The directions captured by the sensor field of view during the whole trajectory is shown in Figure 3. Figure 4 shows the attitude estimation error, which is defined as the principal angle between estimated and true DCM and is small and within the predicted covariance (±3σ) error.

## 4. Optimal Tracking Control

A general scalar nonnegative attitude penalty function is utilized to formulate an optimal feedback control for the spacecraft tracking problem. This new variable yields identical performance index values, regardless of the attitude variables selected. The general final finite-time optimal control formulating is given by minimizing the following performance index [29]:

(79) $$ℑ=\frac{1}{2}\Phi \left(t_{f},\delta \omega \left(t_{f}\right),\delta \zeta \left(t_{f}\right)\right)+\frac{1}{2}\;\int_{t_{0}}^{t_{f}}L\left(\delta \omega ,\delta \zeta ,u,t\right)\;dt,$$

which is subject to $\dot{x}={\left[\delta {\dot{\omega }}^{T}\;\delta {\dot{\zeta }}^{T}\right]}^{T}=f\left(\delta \omega ,\delta \zeta ,u\right)$, where δω is the angular velocity error, δζ is an arbitrary attitude representation of the attitude error, and the penalty functions are (using $\delta \omega \left(t_{f}\right)\;=\;\delta \omega_{t_{f}}$ and $\delta \zeta \left(t_{f}\right)=\delta \zeta_{t_{f}}$):

(80) $$\begin{array}{ccc} \Phi (t_{f},\delta \omega \left(t_{f}\right),\delta \zeta \left(t_{f}\right)) & = & Q_{1}g\left(\delta \zeta_{t_{f}}\right)+\delta \omega_{t_{f}}^{T}Q_{2}\delta \omega_{t_{f}}\\ \mathrm{and}\;\;\;\;\;\;\;\;\;\;\;\;\;L(\delta \omega ,\delta \zeta ,u,t) & = & Q_{3}g\left(\delta \zeta \right)+\delta \omega^{T}Q_{4}\delta \omega +u^{T}Ru. \end{array}$$

The weights $Q_{1}$ and $Q_{3}$ are scalars, and the weights $Q_{2}$, $Q_{4}$, and *R* are 3×3 matrices. The f(δω,δζ,u) is the spacecraft error dynamics. The scalar function g(δζ) is a general nonnegative attitude penalty function. The function is chosen to produce the same cost for a given physical orientation [23,29]:

(81) $$g\left(\left[\delta C\left(\delta \hat{e},\delta \phi \right)\right]\right)=\frac{1}{4}\left(3-\mathrm{tr}\left(\left[\delta C\right]\right)\right)=\sin^{2}\left(\delta \phi /2\right),$$

when using exact nonlinear attitude error kinematics, the orientation will work for large angles; $\delta \phi =\pm 180^{\circ }$. Therefore, the function is bounded 0≤g(δC)≤1 for all possible rotations. Thus, the attitude cost reaches its highest value at the maximum rotation angle. Defining the attitude cost function in terms of the DCM makes it universally valid for arbitrary choice of attitude coordinates. It can be simply parameterized by any other attitude coordinate. The universal quadratic penalty function for arbitrary attitude error representations is given in Table 3. This penalty function returns the same cost for a given physical attitude, thereby eliminating the dependency of the optimal control solution on the choice of the attitude coordinate.

### 4.1. Reference Angular Velocity Defined in the Body Attitude Frame

The reference $\omega_{r}$ and current ω angular velocities are expressed in the same coordinate frames; i.e., $\delta \omega =\omega -\omega_{r}$. It can be shown that the general expression of the attitude error kinematics of this set can be given as follows:

(82) $$\delta \dot{\zeta }=\left[f_{\delta \zeta }\left(\delta \zeta \right)\right]\delta \omega -\left[\;\omega_{r}\times \right]\delta \zeta ,$$

with initial state $\delta \zeta \left(t_{0}\right)=\delta \zeta_{0}$. The Hamiltonian *H* for this system of equations is defined, for the given optimal control problem in Equations (79) and (80), as follows:

(83) $$\begin{array}{cc} H= & \frac{1}{2}\left(Q_{3}g\left(\delta \zeta \right)+\delta \omega^{T}Q_{4}\delta \omega +u^{T}Ru\right)-\\ \; & \lambda_{\delta \omega }^{T}I^{-1}\left(\left[\omega_{r}\times \right]I-\left[\left(I\omega_{r}\right)\times \right]\right)\delta \omega +\left[\delta \omega \times \right]I\delta \omega -u+I\dot{\omega_{r}}+\left[\omega_{r}\times \right]I\omega_{r})+\\ \; & \lambda_{\delta \zeta }^{T}\left(\left[f_{\delta \zeta }\left(\delta \zeta \right)\right]\delta \omega -\left[\;\omega_{r}\times \right]\delta \zeta \right), \end{array}$$

where $\lambda_{\delta \omega }$ and $\lambda_{\delta \zeta }$ are the co-state variables for the angular velocity error and the attitude error, respectively. Invoking the standard necessary condition for optimality, the co-state differential equations are given by the following:

(84) $$\begin{array}{c} {\dot{\lambda }}_{\delta \omega }=-Q_{4}\delta \omega -{\left[f_{\delta \zeta }\left(\delta \zeta \right)\right]}^{T}\lambda_{\delta \zeta }-\left(I\left[\;\delta \omega \times \right]-\left[\;\left(I\delta \omega \right)\times \right]-{\left(\left[\omega_{r}\times \right]I-\left[\left(I\omega_{r}\right)\times \right]\right)}^{T}\right)I^{-1}\lambda_{\delta \omega }\\ {\dot{\lambda }}_{\delta \zeta }=-\frac{1}{2}Q_{3}\frac{\partial g}{\partial (\delta \zeta )}-\frac{\partial }{\partial (\delta \zeta )}{\left(\left[f_{\delta \zeta }\left(\delta \zeta \right)\right]\delta \omega \right)}^{T}\lambda_{\delta \zeta }-\left[\;\omega_{r}\times \right]\lambda_{\delta \zeta }, \end{array}$$

The two co-state differential equations are integrated backward in time with given terminal values $\lambda_{\delta \omega }\left(t_{f}\right)=\frac{\partial \Phi }{\partial (\delta \omega )}{|}_{t_{f}}$ and $\lambda_{\delta \zeta }=\frac{\partial \Phi }{\partial (\delta \zeta )}{|}_{t_{f}}$. The optimal control is given by the first-order necessary conditions for an extremum, $H_{u}=0$, leading to $u=-{\left(IR\right)}^{-1}\lambda_{\delta \omega }$.

### 4.2. Reference Angular Velocity Defined in the Reference Attitude Frame

The reference and current angular velocities are expressed in different coordinate frames, i.e., $\delta \omega =\omega -\delta C\omega_{r}$. Therefore, this definition explicitly computes the angular velocity error in the current body frame. As an important result of this set, the expressions of the attitude error kinematics follow the attitude kinematics equation for any given attitude representation choice. Therefore, the general expression of the attitude error kinematics of this set can be given as follows:

(85) $$\delta \dot{\zeta }=\left[f_{\delta \zeta }\left(\delta \zeta \right)\right]\;\delta \omega ,$$

with initial state $\delta \zeta \left(t_{0}\right)=\delta \zeta_{0}$. The Hamiltonian *H* for this system of equations is as follows:

(86) $$\begin{array}{cc} H= & \frac{1}{2}\left(Q_{3}g\left(\delta \zeta \right)+\delta \omega^{T}Q_{4}\delta \omega +u^{T}Ru\right)-\\ \; & \lambda_{\delta \omega }^{T}I^{-1}(\left(\left[\;\delta C\omega_{r}\times \right]I-\left[\;I\delta C\omega_{r}\times \right]\right)\delta \omega +\left[\;\delta \omega \times \right]I\delta \omega -u-\\ \; & I\left[\;\delta \omega \times \right]\delta C\omega_{r}+I{\dot{\omega }}_{r}+\left[\;\delta C\omega_{r}\times \right]J\delta C\omega_{r})+\lambda_{\delta \zeta }^{T}\left[f_{\delta \zeta }\left(\delta \zeta \right)\right]\delta \omega . \end{array}$$

Invoking the standard necessary condition for optimality, the co-state differential equations are given by the following:

(87) $$\begin{array}{c} {\dot{\lambda }}_{\delta \omega }=-Q_{4}\delta \omega -{\left[f_{\delta \zeta }\left(\delta \zeta \right)\right]}^{T}\lambda_{\delta \zeta }-\left(I\left[\;\delta \omega \times \right]-\left[\;\left(I\delta \omega \right)\times \right]-{\left(\left[\delta C\omega_{r}\times \right]I-\left[\left(I\delta C\omega_{r}\right)\times \right]\right)}^{T}+\left[\;\delta C\omega_{r}\times \right]I\right)I^{-1}\lambda_{\delta \omega }\\ {\dot{\lambda }}_{\delta \zeta }=-\frac{1}{2}Q_{3}\frac{\partial g}{\partial (\delta \zeta )}-\frac{\partial }{\partial (\delta \zeta )}{\left(\left[f_{\delta \zeta }\left(\delta \zeta \right)\right]\delta \omega \right)}^{T}\lambda_{\delta \zeta }-\left[\;\omega_{r}\times \right]\lambda_{\delta \zeta }-\frac{\partial }{\partial (\delta \zeta )}{\left[\delta \dot{}\omega \right]}^{T}\lambda_{\delta \omega }, \end{array}$$

The optimal control is given by the first-order necessary conditions for an extremum, $H_{u}=0$, leading to $u=-{\left(IR\right)}^{-1}\lambda_{\delta \omega }$. Note the last term in the (${\dot{\lambda }}_{\delta \zeta }$) expression involves calculating the partial derivative of the angular velocity error kinematic, $\frac{\partial }{\partial (\delta \zeta )}\left[\delta \dot{}\omega \right]$, which obviously leads to difficult math. This step is also required when performing coordinate mapping between the DCM into other attitude parameters.

### 4.3. Numerical Simulation

**Figure 5:**
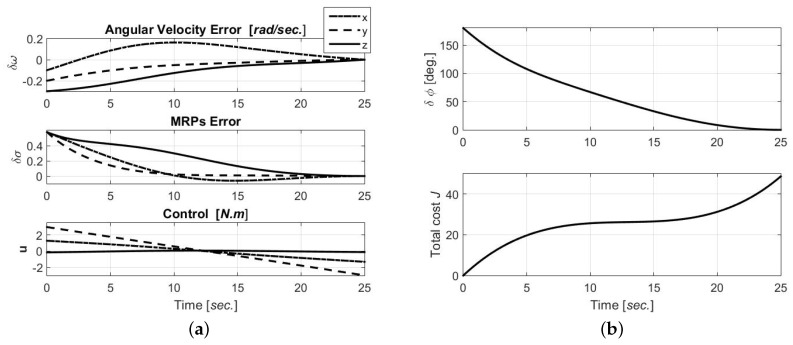
Open-loop optimal solution for the tracking motion.(**a**) Time history of the optimal trajectory, ω and MRPs, and control. (**b**) Time history of the principal angle and the total optimal cost.

This section presents simulation results of a fixed final-time and final-state open-loop optimal control solution for the spacecraft tracking problem. Modified Rodrigues parameters are used for the attitude motion. The initial and final state variable conditions for this example are given in Table 4. The spacecraft moment of inertia tensor is given in Table 5. For simplicity, the weighting matrices are $Q_{1}=0$, $Q_{3}=1$, $Q_{2}=0_{3\times 3}$, and $Q_{4}=R=I_{3\times 3}$. However, one can sweep those penalties to obtain different solutions sets. This example stands to solve the open-loop optimal control problem for the spacecraft tracking problem using arbitrary attitude representation. We consider the universal performance index given in Table 3. The state and co-state differential equations are solved in a Boundary Value Problem (BVP) framework using a shooting method (MATLAB fsolve) [29,33]. The optimal open-loop solution is shown in Figure 5. The time history of the optimal trajectory and control is shown in Figure 5a for MRPs representation. The trajectory is controlled to drive the spacecraft for a given initial state error, δω(0) and δζ(0), to rest at zero attitude error after 25 seconds. It is noted that the optimal open-loop solution obtained for various attitude representation produces the same angular displacement δϕ and total cost, as given in Figure 5b.

## 5. Conclusions

Full analytical derivations of attitude error kinematics equations have been presented. Compact forms of attitude error kinematics equations are derived for various attitude parameterizations. The attitude error is defined as the rotation error between the true and estimated orientations. Several attitude error representations are developed for describing the orientation error kinematics. Two approaches to attitude error kinematics are introduced. The first one considers the estimated angular velocity defined in the true body axes, while in the second one, the estimated angular velocity is defined in the estimated body axes. These two angular velocity definitions are usually adopted in simulations and in real estimation/control applications, respectively. These two nonlinear kinematic models are valid for arbitrarily large relative rotations and rotation rates. These results are expected to be broadly useful for generalizing extended Kalman filtering formulations and optimal control tracking problems.

## Figures and Tables

**Table 1:** Initial conditions.

Parameter	Initial Condition
ω (rad/s)	${\left\{0.7972,\;0.5202,\;0.3064\right\}}^{T}$
$\hat{\omega }$ (rad/s)	${\left\{0.7931,\;0.5898,\;0.1521\right\}}^{T}$
q	${\left\{-0.3112,\;-0.2937,\;0.5374,\;-0.7267\right\}}^{T}$
$\hat{q}$	${\left\{-0.0104,\;-0.8248,\;0.4319,\;0.3647\right\}}^{T}$

**Table 2:** The extended Kalman filter (EKF) model.

$\delta \omega =\omega -\hat{\omega }$	$\delta \omega =\omega -\delta C\hat{\omega }$
$\begin{array}{cc} F & =\left[\begin{array}{cc} -[\hat{\omega }\times ] & -\frac{1}{2}I_{3\times 3}\\ 0_{3\times 3} & 0_{3\times 3} \end{array}\right]\\ G & =\left[\begin{array}{cc} -\frac{1}{2}I_{3\times 3} & 0_{3\times 3}\\ 0_{3\times 3} & I_{3\times 3} \end{array}\right]\\ Q & =\left[\begin{array}{cc} \sigma_{v}^{2}I_{3\times 3} & 0_{3\times 3}\\ 0_{3\times 3} & \sigma_{v}^{2}I_{3\times 3} \end{array}\right] \end{array}$	$\begin{array}{cc} F & =\left[\begin{array}{cc} -[\left(\hat{\omega }+\hat{\beta }\right)\times ] & -\frac{1}{2}I_{3\times 3}\\ 2\left[\hat{\beta }\times \right]\left[\left(\hat{\omega }+\hat{\beta }\right)\times \right] & \left[\hat{\beta }\times \right] \end{array}\right]\\ G & =\left[\begin{array}{cc} -\frac{1}{2}I_{3\times 3} & 0_{3\times 3}\\ \hat{\beta }\times ] & I_{3\times 3} \end{array}\right]\\ Q & =\left[\begin{array}{cc} \sigma_{v}^{2}I_{3\times 3} & 0_{3\times 3}\\ 0_{3\times 3} & \sigma_{v}^{2}I_{3\times 3} \end{array}\right] \end{array}$

**Table 3:** Universal penalty function for attitude error.

Attitude Parameter	Penalty Function
DCM	$g\left(\delta C\right)=\frac{1}{4}\left(3-\mathrm{tr}\left(\delta C\right)\right)$
Quaternion	$g\left(\delta C\left(\delta q\right)\right)=\delta q_{1}^{2}+\delta q_{2}^{2}+\delta q_{3}^{2}$
CRPs	$g\left(\delta C\left(\delta \rho \right)\right)=\frac{\delta \rho^{T}\delta \rho }{1+\delta \rho^{T}\delta \rho }$
MRPs	$g\left(\delta C\left(\delta \sigma \right)\right)=4\frac{\delta \sigma^{T}\delta \sigma }{{\left(1+\delta \sigma^{T}\delta \sigma \right)}^{2}}$
Euler Angles${}^{*}$	$g\left(\delta C\left(\delta \theta \right)\right)=\frac{1}{4}\left[3-\left(1+\cos\delta \theta_{2}\right)\cos\left(\delta \theta_{1}+\delta \theta_{3}\right)-\cos\delta \theta_{2}\right]$
Principal angle/axis	$g\left(\delta C\left(\delta \hat{e},\delta \phi \right)\right)=\sin^{2}\left(\delta \phi /2\right)$
Cayley-Klein	$g\left(\delta C\left(\delta K\right)\right)=1-\frac{1}{4}\mathrm{tr}{\left(\delta K\right)}^{2}$

${}^{*}$ For the 3-1-3 rotation sequence.

**Table 4:** Initial/boundary conditions.

Time (s)	Attitude Error (MRPs) δζ	Angular Velocity Error δω (rad/s)
0	$\frac{1}{\sqrt{3}}{\left[1,\;1,\;1\right]}^{T}$	${\left[-0.1,\;-0.2,\;-0.3\right]}^{T}$
25	$\;\;\;\;\;{\left[0,\;0,\;0\right]}^{T}$	${\left[0,\;0,\;0\right]}^{T}$

**Table 5:** The spacecraft principal moment of inertia components (kg·m${}^{2}$).

$I_{1}$	86.215
$I_{2}$	85.070
$I_{3}$	113.565

## Appendix A. Angular Velocity Error Vector and Euler Angle Error Rates Mapping

This appendix presents forward and inverse mapping between the angular velocity error vector δω and the Euler angle error rates $\delta \dot{\theta }={\left\{\begin{array}{ccc} \delta {\dot{\theta }}_{1} & \delta {\dot{\theta }}_{2} & \delta {\dot{\theta }}_{3} \end{array}\right\}}^{T}$ in the following form:

$$\delta \dot{\theta }=M_{ijk}^{-1}\;\delta \omega$$

Note that the shorthand notations $c_{i}=\cos\left(\delta \theta_{i}\right)$ and $s_{i}=\sin\left(\delta \theta_{i}\right)$ are used in Table A1.

**Table A1:** Angular velocity error vector and Euler angle error rates mapping.

*i*-*j*-*k*	$M_{\mathrm{ijk}}^{-1}$	$M_{\mathrm{ijk}}$	*i*-*j*-*k*	$M_{\mathrm{ijk}}^{-1}$	$M_{\mathrm{ijk}}$
1-2-1	$\frac{1}{s_{2}}\left[\begin{array}{ccc} 0 & s_{3} & c_{3}\\ 0 & s_{2}c_{3} & -s_{2}c_{3}\\ s_{2} & -c_{2}s_{3} & -c_{2}c_{3} \end{array}\right]$	$\left[\begin{array}{ccc} c_{2} & 0 & 1\\ s_{2}s_{3} & c_{3} & 0\\ s_{2}c_{3} & -s_{3} & 0 \end{array}\right]$	2-3-1	$\frac{1}{c_{2}}\left[\begin{array}{ccc} 0 & c_{3} & -s_{3}\\ 0 & c_{2}s_{3} & c_{2}c_{3}\\ c_{2} & -s_{2}c_{3} & s_{2}s_{3} \end{array}\right]$	$\left[\begin{array}{ccc} s_{2} & 0 & 1\\ c_{2}c_{3} & s_{3} & 0\\ -c_{2}s_{3} & c_{3} & 0 \end{array}\right]$
					
1-2-3	$\frac{1}{c_{2}}\left[\begin{array}{ccc} c_{3} & -s_{3} & 0\\ c_{2}s_{3} & c_{2}c_{3} & 0\\ -s_{2}c_{3} & s_{2}s_{3} & c_{2} \end{array}\right]$	$\left[\begin{array}{ccc} c_{2}c_{3} & s_{3} & 0\\ -c_{2}s_{3} & c_{3} & 0\\ s_{2} & 0 & 1 \end{array}\right]$	2-3-2	$\frac{1}{s_{2}}\left[\begin{array}{ccc} c_{3} & 0 & s_{3}\\ -s_{2}s_{3} & 0 & s_{2}c_{3}\\ -c_{2}c_{3} & s_{2} & -c_{2}s_{3} \end{array}\right]$	$\left[\begin{array}{ccc} s_{2}c_{3} & -s_{3} & 0\\ c_{2} & 0 & 1\\ s_{2}s_{3} & c_{3} & 0 \end{array}\right]$
					
1-3-1	$\frac{1}{s_{2}}\left[\begin{array}{ccc} 0 & -c_{3} & s_{3}\\ 0 & s_{2}s_{3} & s_{2}c_{3}\\ s_{2} & c_{2}c_{3} & -c_{2}s_{3} \end{array}\right]$	$\left[\begin{array}{ccc} c_{2} & 0 & 1\\ -s_{2}c_{3} & s_{3} & 0\\ s_{2}s_{3} & c_{3} & 0 \end{array}\right]$	3-1-2	$\frac{1}{c_{2}}\left[\begin{array}{ccc} -s_{3} & 0 & c_{3}\\ c_{2}c_{3} & 0 & c_{2}s_{3}\\ s_{2}s_{3} & c_{2} & -s_{2}c_{3} \end{array}\right]$	$\left[\begin{array}{ccc} -c_{2}s_{3} & c_{3} & 0\\ s_{2} & 0 & 1\\ c_{2}c_{3} & s_{3} & 0 \end{array}\right]$
					
1-3-2	$\frac{1}{c_{2}}\left[\begin{array}{ccc} c_{3} & 0 & s_{3}\\ -c_{2}s_{3} & 0 & c_{2}c_{3}\\ s_{2}c_{3} & c_{2} & s_{2}s_{3} \end{array}\right]$	$\left[\begin{array}{ccc} c_{2}c_{3} & -s_{3} & 0\\ -s_{2} & 0 & 1\\ c_{2}s_{3} & c_{3} & 0 \end{array}\right]$	3-1-3	$\frac{1}{s_{2}}\left[\begin{array}{ccc} s_{3} & c_{3} & 0\\ s_{2}c_{3} & -s_{2}s_{3} & 0\\ -c_{2}s_{3} & -c_{2}c_{3} & s_{2} \end{array}\right]$	$\left[\begin{array}{ccc} s_{3}s_{2} & c_{3} & 0\\ s_{2}c_{3} & -s_{3} & 0\\ c_{2} & 0 & 1 \end{array}\right]$
					
2-1-2	$\frac{1}{s_{2}}\left[\begin{array}{ccc} s_{3} & 0 & -c_{3}\\ s_{2}c_{3} & 0 & s_{2}s_{3}\\ -c_{2}s_{3} & s_{2} & c_{2}c_{3} \end{array}\right]$	$\left[\begin{array}{ccc} s_{2}s_{3} & c_{3} & 0\\ c_{2} & 0 & 1\\ -s_{2}c_{3} & s_{3} & 0 \end{array}\right]$	3-2-1	$\frac{1}{c_{2}}\left[\begin{array}{ccc} 0 & 0 & c_{3}\\ 0 & c_{2}c_{3} & -c_{2}s_{3}\\ c_{2} & s_{2}s_{3} & s_{2}c_{3} \end{array}\right]$	$\left[\begin{array}{ccc} -s_{2} & 0 & 1\\ c_{2}s_{3} & c_{3} & 0\\ c_{2}c_{3} & -s_{3} & 0 \end{array}\right]$
					
2-1-3	$\frac{1}{c_{2}}\left[\begin{array}{ccc} s_{3} & c_{3} & 0\\ c_{2}c_{3} & -c_{2}s_{3} & 0\\ s_{2}s_{3} & s_{2}c_{3} & c_{2} \end{array}\right]$	$\left[\begin{array}{ccc} c_{2}s_{3} & c_{3} & 0\\ c_{2}c_{3} & -s_{3} & 0\\ -s_{2} & 0 & 1 \end{array}\right]$	3-2-3	$\frac{1}{s_{2}}\left[\begin{array}{ccc} -c_{3} & s_{3} & 0\\ s_{2}s_{3} & s_{2}c_{3} & 0\\ c_{2}c_{3} & -c_{2}s_{3} & s_{2} \end{array}\right]$	$\left[\begin{array}{ccc} -s_{2}c_{3} & s_{3} & 0\\ s_{2}s_{3} & c_{3} & 0\\ c_{2} & 0 & 1 \end{array}\right]$
					

## Appendix B. Attitude Error Kinematics

Attitude error kinematics equations for various attitude representations and for the two approaches—estimated angular velocity defined in the true attitude frame (for simulations) or defined in the estimated attitude frame (for real applications)—are summarized in Figure A1. These equations are used to integrate the attitude error rate.
